# PARP Inhibitors and the Risk of Serum Creatinine Elevation in Ovarian Cancer: A Systematic Review and Meta-Analysis of Randomized Controlled Trials

**DOI:** 10.3390/cancers18081226

**Published:** 2026-04-13

**Authors:** Agnieszka Gąsowska-Bodnar, Beata Gąsowska-Bajger, Aleksandra Żołnierek, Jakub Żołnierek, Lubomir Bodnar

**Affiliations:** 1 Institute of Medical and Health Sciences, University of Siedlce, 08-110 Siedlce, Poland; 2 Institute of Chemistry, Opole University, 45-052 Opole, Poland; 3 Department of Dermatology, Military Institute of Medicine-National Research Institute, Central Clinical Hospital Ministry of Defence, 04-141 Warsaw, Poland

**Keywords:** PARP inhibitors, ovarian cancer, nephrotoxicity, creatinine increase

## Abstract

Background: Poly(ADP-ribose) polymerase inhibitors (PARPis) are established maintenance therapies in epithelial ovarian cancer (EOC). Although considered relatively safe, their impact on renal function remains unclear. Increases in serum creatinine (SCr) are frequently observed during treatment, but the clinical significance of these changes is uncertain. We conducted a systematic review and meta-analysis to assess the risk of renal adverse events associated with PARPis in randomized controlled trials (RCTs). Methods: PubMed/MEDLINE, Embase, and the Cochrane Library were searched for phase II–III, placebo-controlled RCTs published through 30 June 2025. Eligible studies enrolled patients with ovarian cancer receiving maintenance monotherapy with olaparib, niraparib, rucaparib, or fuzuloparib and reported renal adverse events. The primary endpoint was creatinine increase (all grades). Pooled odds ratios (ORs) with 95% confidence intervals (CIs) were calculated using fixed- or random-effects models according to heterogeneity. Results: Nine high-quality RCTs comprising 2578 patients met the inclusion criteria. PARPi therapy was associated with a significantly increased risk of creatinine elevation compared with placebo (OR 5.04; 95% CI 3.51–7.24; *p* < 0.001). Heterogeneity was moderate (I^2^ = 31.7%). High-grade renal adverse events (≥grade 3) were rare (<1%) and could not be reliably pooled. No significant publication bias was detected. Conclusions: PARP inhibitors significantly increase the likelihood of SCr elevation in EOC; however, severe nephrotoxicity appears uncommon in RCTs. Observed SCr increases may partly reflect inhibition of renal tubular creatinine transport rather than true reductions in glomerular filtration. Careful renal monitoring and prospective studies incorporating direct GFR assessment are warranted.

## Simple Summary

PARP inhibitors are a cornerstone of maintenance therapy in ovarian cancer, yet their effects on kidney function remain incompletely understood. Increases in serum creatinine are frequently observed during treatment and may raise concerns about drug-induced nephrotoxicity, potentially influencing clinical decision-making. However, these changes may reflect drug-related interference with renal creatinine handling rather than true kidney injury. In this systematic review and meta-analysis of randomized controlled trials, PARP inhibitors were associated with a significantly increased risk of creatinine elevation, while severe, clinically significant nephrotoxicity remained rare. These findings highlight potential discrepancies between laboratory markers and true renal function and reveal limitations of current monitoring strategies. Our results support the need for more accurate assessment of kidney function beyond serum creatinine to avoid unnecessary treatment modifications and to optimize long-term patient management.

## 1. Introduction

Epithelial ovarian cancer (EOC) remains one of the most lethal malignancies affecting women, ranking as the fifth leading cause of cancer-related mortality worldwide [1]. Approximately 20–25% of cases arise in the context of hereditary cancer syndromes, most commonly due to pathogenic BRCA1 or BRCA2 variant populations [2]. The introduction of poly(ADP-ribose) polymerase inhibitors (PARPis) has markedly transformed the management of EOC. PARP inhibitors have demonstrated substantial efficacy across multiple phase III clinical trials, producing marked improvements in progression-free survival both in BRCA-mutated and BRCA-wild-type populations, with hazard ratios of 0.32 and 0.57, respectively [3]. Their therapeutic activity is grounded in the principle of synthetic lethality: inhibition of PARP1 results in the accumulation of DNA single- and double-strand breaks, which become lethal in cells deficient in homologous recombination repair—most notably those harboring BRCA1/2 pathogenic variants [4]. Although PARP inhibitors have been largely perceived as safe and well tolerated, their potential impact on renal function remains insufficiently characterized. Preclinical data suggest a paradoxical picture. On the one hand, PARP-1 activation has been implicated in the pathogenesis of acute kidney injury (AKI), particularly in the setting of ischemia–reperfusion injury. In murine experiments, genetic ablation of PARP-1 attenuated tubular damage, preserved ATP stores, mitigated inflammatory responses, and improved functional renal outcomes, indicating that excessive PARP signalling may potentiate acute tubular injury [5]. Experimental data, in turn, show that PARPis can inhibit renal tubular creatinine secretion without affecting true glomerular filtration rate, leading to an apparent decline in estimated GFR (eGFR) despite stable measured GFR [6]. Clinically, a small cohort study of ovarian cancer patients showed that approximately one-third experienced an early decline in eGFR shortly after initiating niraparib treatment; most cases stabilized, but three patients developed persistent renal failure of unclear etiology [7]. A retrospective study by Gupta and colleagues in 2023, including 269 patients with ovarian cancer treated with PARP inhibitors (olaparib or niraparib), assessed the impact of therapy on renal function. Acute kidney injury (AKI) occurred in 22.3% of patients within 12 months, with similar rates for both drugs. However, only 3.3% of cases (nine) were directly attributed to PARPis; most AKI were mild (91.7% KDIGO stage 1). Persistent AKI was reported in 35% of cases (7.8% of the cohort), of which only 2.2% were attributed to PARPis [8].

However, solid evidence remains lacking, as no large, long-term studies have comprehensively examined the course of renal function in patients treated with PARPis using reliable markers beyond serum creatinine. Given these uncertainties, we conducted a meta-analysis focused on assessing the renal safety of PARP inhibitor therapy. This study included a systematic literature review and a meta-analysis of phase II and III randomized trials to comprehensively evaluate the incidence and relative risk (RR) of nephrotoxicity in patients receiving PARPi-based treatment for EOC.

## 2. Methods

### 2.1. Search Strategy

We systematically searched Embase, PubMed/MEDLINE, and the Cochrane Library for randomized controlled trials (RCTs) using the following terms: *“PARPi,” “PARP inhibitor,” “olaparib,” “niraparib,” “rucaparib,” “talazoparib,”, “fuzuloparib”, “nephrotoxicity,” “renal toxicity,” “acute kidney injury,” “creatinine increase,”* and *“glomerular filtration rate.”* The search was limited to English-language publications available up to 30 June 2025. Additionally, we reviewed abstracts from the ASCO and ESMO annual meetings. Reference lists of eligible articles and relevant reviews were manually screened to identify additional studies. When multiple publications of the same trial were available, the most recent and most complete version was selected. The study protocol was prospectively registered in the PROSPERO database (registration number: CRD42024505697).

### 2.2. Inclusion and Exclusion Criteria

Studies were included if they met the following criteria: (1) enrolled patients with ovarian cancer; (2) were prospective phase II or III RCTs; (3) compared PARPi maintenance monotherapy with a placebo control; and (4) reported renal adverse events (AEs) in PARPi-treated versus non-PARPi-treated patients, including nephrotoxicity (e.g., acute kidney injury [AKI], renal failure), creatinine elevation (≥1.5× baseline), or estimated glomerular filtration rate (eGFR) decline (≥30% from baseline), classified according to CTCAE v5.0 as all-grade (1–5) or high-grade (3–5).

Exclusion criteria included: phase I studies; single-arm trials; studies without renal AEs reported in either arm; trials involving concomitant nephrotoxic therapies without clear attribution (e.g., platinum-based chemotherapy); retrospective or observational studies; meeting abstracts without full data; case reports; incomplete trials; duplicate publications; letters; reviews; and articles published in languages other than English.

### 2.3. Study Selection

Two investigators (AZ, BGB) independently screened titles, abstracts, and full texts according to the PICO framework (Population, Intervention, Comparison, Outcome). Discrepancies were resolved by consensus or consultation with a third reviewer (LB). The quality of the included studies was independently assessed using the 5-point Jadad scale (evaluating randomization, blinding, and withdrawals/dropouts; score range 0–5) separate from effect size estimation.

### 2.4. Data Extraction

Two authors (AZ, BGB) independently extracted data in accordance with PRISMA guidelines, resolving disagreements through discussion. Extracted variables included: first author, publication year, study phase, treatment arms, primary endpoints, tumor type, sample size, comparator, and renal AEs (all-grade and high-grade AKI, creatinine increase, eGFR decrease, and other nephrotoxic events).

### 2.5. Data Analysis

Odds ratios (ORs) with 95% confidence intervals (CIs) were pooled using fixed- or random-effects models (Mantel–Haenszel) when heterogeneity was low (I^2^ < 50%) or random-effects models (DerSimonian–Laird) when heterogeneity was high. Statistical heterogeneity was quantified using the Q test and I^2^ statistic. Sensitivity analyses were performed by sequential study exclusion. Publication bias was assessed using funnel plots. All analyses were conducted using Stata version 16.0, with two-sided *p*-values < 0.05 considered statistically significant.

## 3. Results

### 3.1. Literature Search

The literature search identified 12,195 potentially relevant clinical trials evaluating PARP inhibitors (PARPis) across solid tumors with relevance to epithelial ovarian cancer (EOC). After removal of duplicates from PubMed, ACS, MEDLINE, and Scopus and screening of titles and abstracts, 11,915 records were excluded as they did not meet the inclusion criteria. These comprised duplicates, narrative reviews, meta-analyses, systematic reviews, and preclinical studies.

**Figure 1:**
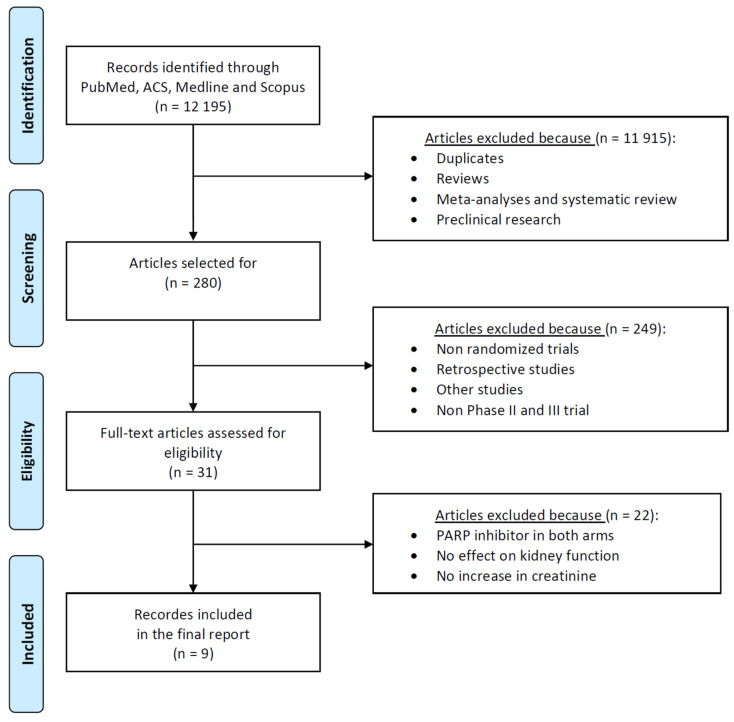
PRISMA 2009 flow diagram illustrating study selection [9].

Full-text assessment was performed for 280 articles, of which 249 were excluded due to non-randomized or retrospective design, inappropriate study phase, or other ineligible designs. Of the remaining 31 articles, 22 were excluded because PARP inhibitors were used in both arms, renal outcomes were not assessed, or creatinine data were unavailable (Figure 1).

Ultimately, nine randomized controlled trials (RCTs) met the eligibility criteria and were included in the meta-analysis. All studies enrolled patients with high-grade epithelial ovarian cancer and investigated approved PARP inhibitors: olaparib (two trials), niraparib (four trials), rucaparib (two trials), and fuzuloparib (one trial). In total, 2578 patients were included. All trials evaluated maintenance therapy, with placebo as the comparator.

### 3.2. Characteristics of Included Studies

The analysis comprised nine phase II–III randomized, placebo-controlled trials, including 2578 patients with ovarian cancer treated in a maintenance setting. Five trials enrolled patients with platinum-sensitive recurrent disease (ARIEL3; Study-19; NOVA; NORA; FZOCUS-2), while four trials focused on newly diagnosed advanced ovarian cancer (PRIMA; SOLO-2; PRIME; ATHENA).

Across studies, progression-free survival (PFS) was consistently reported as the primary endpoint. Niraparib was evaluated in four trials, olaparib and rucaparib in two trials each, and fuzuloparib in one trial. Methodological quality was uniformly high, with all studies achieving a Jadad score of 5. Key characteristics of the included trials are summarized in Table 1.

### 3.3. Risk of Creatinine Level Increase Associated with PARP Inhibitors

Across nine randomized trials, treatment with PARP inhibitors was associated with a significantly increased risk of creatinine level elevation compared with control arms. High-grade (≥grade 3) increases in creatinine level were not pooled due to their extremely low incidence across studies, which resulted in unstable estimates and excessively wide confidence intervals. The pooled analysis demonstrated a strong and statistically significant association, with an overall odds ratio (OR) of 5.04 (95% CI, 3.51–7.24), indicating a more than five-fold higher likelihood of creatinine increase among patients receiving PARP inhibitors.

Individual study estimates consistently favored the control arms, although the magnitude of effect varied across trials. Between-study heterogeneity was moderate (I^2^ = 31.7%, H^2^ = 1.46), and the Cochran Q test did not reveal statistically significant heterogeneity (Q = 11.71, *p* = 0.16). The overall pooled effect was highly significant (Z = 8.77, *p* < 0.001).

**Figure 2:**
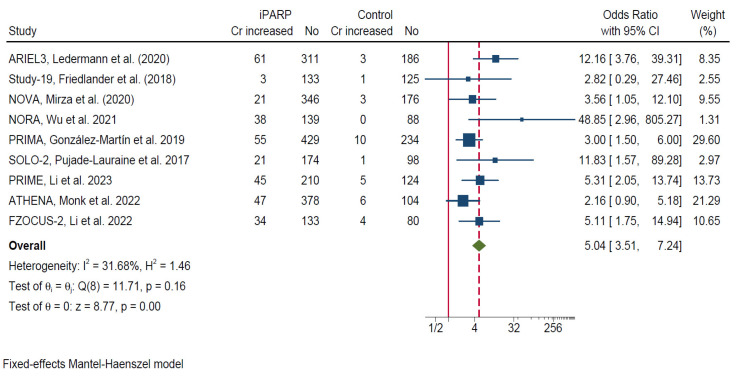
Odds ratio for creatinine level increase in patients treated with PARP-inhibitor-based therapies compared with control. Abbreviation: CI, confidence interval; Cr, creatinine; df, degrees of freedom; OR, odds ratio; PARP, poly(ADP-ribose) polymerase; PARPi, PARP inhibitor. Ledermann, J.A.; et al. (2020) [9]; Friedlander, M.; et al. (2018) [10]; Mirza, M.R.; et al. (2020) [11]; Wu, X.H.; et al. 2021 [12]; González-Martín, A.; et al. 2019 [13]; Pujade-Lauraine, E.; et al. 2017 [14]; Li, N.; et al. 2023 [15]; Monk, B.J.; et al. 2022 [16]; Li, N.; et al. 2022 [17].

Given the limited heterogeneity and methodological consistency across studies, a fixed-effects Mantel–Haenszel model was applied. Sensitivity analysis with a random-effects (DerSimonian–Laird) model produced a comparable estimate (OR = 4.10, 95% CI, 2.80–6.00; *p* < 0.001), indicating robust and consistent results across modelling approaches. These findings indicate that PARP inhibitor therapy is consistently associated with an increased risk of creatinine elevation across different clinical settings in ovarian cancer (Figure 2).

### 3.4. Publication Bias

**Figure 3:**
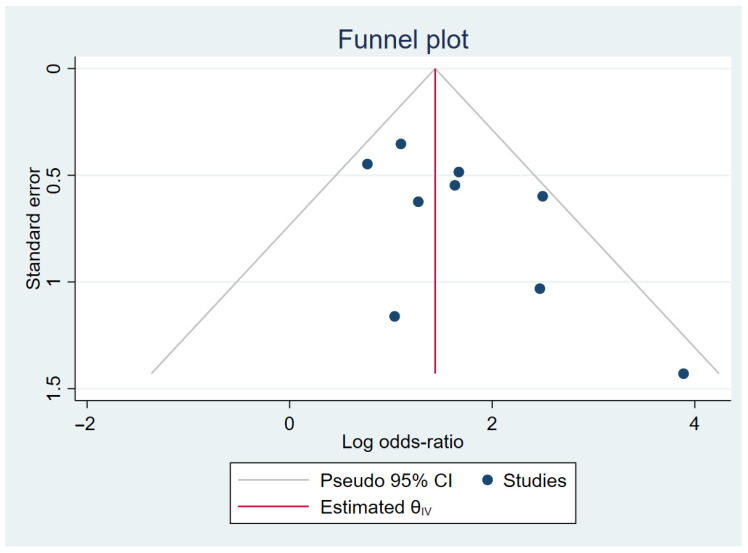
Funnel plots for increased creatinine and use of PARP inhibitors.

We used Begg’s test to estimate publication bias in our meta-analysis. We did not observe for publication bias in the Begg’s test between increased creatinine and use of PARPis in patients with ovarian carcinoma (Figure 3).

## 4. Discussion

Our study is the first metanalysis to assess and compare the relationship between PARPi therapies and ARF in randomized clinical trials II, III phases.

Analysis of FDA Adverse Event Reporting System (FAERS) data from 2015–2023 confirmed a significant association between the use of selected PARP inhibitors and the occurrence of acute renal failure (ARF). Olaparib, niraparib and rucaparib demonstrated statistically significant safety signals (reporting odds ratios: 1.62, 1.25 and 1.59, respectively), whereas talazoparib showed no such association. In total, 2726 ARF cases were identified, and the median time to onset ranged from 36 to 85 days, indicating a potentially delayed pattern of toxicity. Notably, the highest mortality rate was observed in patients treated with olaparib (9.88%), while niraparib was more frequently associated with reported life-threatening adverse events (4.89%) [18,19].

Epithelial ovarian cancer (EOC) remains the most lethal gynecologic malignancy owing to its high death-to-incidence ratio. The introduction of PARP inhibitors—particularly olaparib, niraparib and rucaparib—has fundamentally reshaped the therapeutic landscape of ovarian cancer [20]. Olaparib, rucaparib and niraparib are approved as first-line maintenance monotherapies, and olaparib combined with bevacizumab provides additional benefit in newly diagnosed, advanced, homologous recombination-deficient (HRD) ovarian cancer [21]. In the recurrent platinum-sensitive setting, multiple studies support the use of PARP inhibitors as second-line or later maintenance therapy [22]. Although patients with BRCA mutations/HRD-positive cases derive the greatest benefit, those with HRP/BRCA-wild-type tumors may also experience clinical benefit, albeit with substantially lower efficacy [22]. To address PARP inhibitor resistance and broaden therapeutic benefit beyond BRCA-mutated disease, combination strategies involving anti-angiogenic agents, immune checkpoint inhibitors and antibody–drug conjugates are currently under intensive investigation [23].

Accurate assessment and preservation of renal function remain essential at every stage of ovarian cancer management. Serum creatinine (SCr) is widely used as a standard biomarker of glomerular filtration rate (GFR) and serves as a basis for adjusting the doses of renally eliminated drugs. Creatinine is generated primarily in skeletal muscle through the nonenzymatic dehydration and cyclization of creatine and phosphocreatine. Creatine, a nitrogenous organic acid synthesized in the liver, kidneys and pancreas, is phosphorylated to phosphocreatine by creatine kinase (CK) in approximately 75% of the total creatine pool, with the remainder circulating in its free form. Approximately 1.7% of the total body creatine content (1.1% of creatine and 2.6% of phosphocreatine per day) undergoes nonenzymatic conversion to creatinine daily [24].

An increase in serum creatinine (SCr) after administration of a study drug may raise concerns about drug-induced renal impairment and can prompt dose modification or treatment discontinuation [25]. However, SCr levels are influenced not only by glomerular filtration but also by tubular secretion mediated by membrane-bound drug transporters. These include solute carrier (SLC) transporters—such as OATs, OATPs, OCTs, OCTNs, PEPTs and MATEs—and ATP-binding cassette (ABC) transporters, including P-gp, MRPs and BCRP [26,27]. Key renal SLC transporters, notably OCT2, OAT2, OAT3, OCTN1, MATE1 and MATE2-K, coordinate basolateral uptake and apical efflux of creatinine and many xenobiotics [28,29]. Because investigational drugs may inhibit these pathways, SCr elevations can reflect transporter blockade rather than true reductions in GFR, underscoring the need for careful interpretation in pharmacokinetic and safety assessments [30].

Creatinine is actively secreted in the proximal renal tubules, with approximately 10–40% of its clearance mediated by transporters such as MATE1, MATE2-K, OCT2 and OAT2 [31]. PARP inhibitors (PARPis) interact extensively with these renal and hepatic transport pathways, as many agents in this class serve as transporter substrates [32,33]. All clinically available PARPis are substrates of the efflux transporters P-glycoprotein (P-gp) and breast cancer resistance protein (BCRP) [34], and several additionally act as transporter inhibitors [33]. Olaparib inhibits a broad spectrum of transporters, including P-gp, BCRP, OATP1B1, OCT1, OCT2, OAT3, MATE1 and MATE2-K [32]. Niraparib inhibits MATE1 and MATE2-K but does not inhibit BCRP or OCT1 [33]. Rucaparib demonstrates the broadest inhibitory profile, targeting P-gp, BCRP, OATP1B1, OATP1B3, OAT1, OAT3, MATE1, MATE2-K, OCT1, OCT2 and MRP4 [34]. Because PARPis may inhibit one or more of these transport pathways, elevations in serum creatinine may reflect transporter blockade rather than true reductions in glomerular filtration, underscoring the need for careful interpretation in pharmacokinetic and safety assessments [25]. Serum creatinine is an imperfect marker of renal function, as PARP inhibitors can inhibit tubular creatinine secretion, leading to apparent elevations without true GFR impairment. Alternative measures, such as cystatin C or direct GFR assessment (e.g., isotopic methods), may provide more accurate evaluation. Future studies incorporating these biomarkers are warranted to better distinguish functional changes from genuine nephrotoxicity.

Although all PARP inhibitors share a common mechanism of action, they differ in their pharmacological profiles and interactions with renal transporters, which may influence the degree of creatinine elevation. Due to the limited number of studies per individual agent and inconsistent reporting of renal outcomes, formal subgroup analyses were not feasible. Nevertheless, available data suggest that these effects may vary across agents and warrant further investigation. The Japanese Adverse Drug Event Reporting (JADER) database was analyzed to investigate adverse reactions to PARP inhibitors (PARPis), and renal dysfunction signals were confirmed following niraparib treatment. In a Japanese cohort, niraparib therapy was associated with a mean decline in estimated glomerular filtration rate (eGFR) of 28%, and 18% of patients developed acute kidney disease. Notably, this study did not find evidence of tubular injury based on urinary retinol-binding protein testing, although the assessment was limited to only five patients [7]. It should also be noted that analyses based on spontaneous reporting systems such as FAERS and JADER are subject to inherent limitations, including reporting bias, lack of denominator data, and inability to establish causality. A retrospective study examined discrepancies between calculated and measured glomerular filtration rate (GFR) in patients receiving PARP inhibitors who developed elevated serum creatinine. Of 211 patients with ovarian or endometrial cancer, 64 had increased creatinine levels, and 23 underwent technetium-99 m GFR scintigraphy. Despite a median 49% increase in serum creatinine and a significant decrease in calculated creatinine clearance, measured GFR on renography remained essentially unchanged from baseline. A discrepancy between a low creatinine-based estimated GFR and a normal GFR measured by renal scintigraphy occurred in 63% of isotopic studies, suggesting a frequent overestimation of renal dysfunction when creatinine-based formulas are used alone. These findings suggest that the increase in creatinine during PARP inhibitor therapy often reflects extrarenal mechanisms and may not indicate a true impairment of glomerular filtration [6].

In this meta-analysis, we did not perform a separate pooling of high-grade (≥CTCAE grade 3) or serious increases in blood creatinine, as such events were extremely rare in the included RCTs. Across the nine trials, grade 3 or higher renal adverse events occurred in <1% of patients in the PARPi arms (mostly 0–0.6%), often affecting only isolated individuals, with zero or near-zero events in many placebo arms. This very low event rate precluded reliable meta-analytic estimation of relative risk due to instability and excessively wide confidence intervals. While real-world data (FAERS, JADER) report occasional cases of delayed acute renal failure with potential severity, severe clinically significant nephrotoxicity remained exceptional in controlled phase III settings [7,19].

Our meta-analysis of RCTs demonstrated a significantly increased risk of creatinine elevation with PARP inhibitors. Although such rises in SCr during PARPi therapy are often considered reversible and transporter-mediated rather than indicative of true kidney injury, the results of our analysis—as well as real-world data, including FAERS—suggest a less reassuring picture. These findings underscore the importance of careful renal monitoring during PARPi treatment and the need to distinguish benign, transporter-related SCr elevations from genuine nephrotoxicity. Prospective studies incorporating direct and accurate measurements of GFR are particularly warranted, especially for agents used in long-term maintenance therapy, which are generally expected to have lower toxicity and prolonged exposure durations.

## 5. Conclusions

In conclusion, PARP inhibitors significantly increase the risk of serum creatinine elevation, while clinically relevant nephrotoxicity remains uncommon. These findings highlight a potential mismatch between standard laboratory markers and true renal function, with important implications for clinical decision-making. Improved assessment strategies beyond serum creatinine are needed to avoid misinterpretation of renal toxicity and to optimize long-term management in patients receiving PARP inhibitors.

## Figures and Tables

**Table 1:** Characteristics of the included studies.

Trial	Phase	Disease Setting	Treatment Arms	Pts (N)	Control: Placebo (N)	Primary Endpoint	Jadad
ARIEL3, Ledermann, J.A.; et al. (2020) [9]	III	Platinum-sensitive recurrent ovarian cancer	Rucaparib	372	189	PFS	5
Study-19, Friedlander, M.; et al. (2018) [10]	II	Platinum-sensitive recurrent ovarian cancer	Olaparib	136	126	PFS	5
NOVA, Mirza, M.R.; et al. (2020) [11]	III	Platinum-sensitive recurrent ovarian cancer	Niraparib	367	179	PFS	5
NORA, Wu, X.H.; et al. 2021 [12]	III	Platinum-sensitive recurrent ovarian cancer	Niraparib	177	88	PFS	5
PRIMA, González-Martín, A.; et al. 2019 [13]	III	Newly diagnosed advanced ovarian cancer	Niraparib	484	244	PFS	5
SOLO-2, 14. Pujade-Lauraine, E.; et al. 2017 [14]	III	Newly diagnosed advanced ovarian cancer	Olaparib	195	99	PFS	5
PRIME, Li, N.; et al. 2023 [15]	III	Newly diagnosed advanced ovarian cancer	Niraparib	255	129	PFS	5
ATHENA, Monk, B.J.; et al. 2022 [16]	III	Newly diagnosed advanced ovarian cancer	Rucaparib	425	110	PFS	5
FZOCUS-2, Li, N.; et al. 2022 [17]	III	Platinum-sensitive recurrent ovarian cancer	Fuzuloparib	167	84	PFS	5

## Data Availability

The original contributions presented in this study are included in the article. Further inquiries can be directed to the corresponding author(s).

## References

[1] Bray F., Laversanne M., Sung H., Ferlay J., Siegel R.L., Soerjomataram I., Jemal A. (2024). Global Cancer Statistics 2022: GLOBOCAN Estimates of Incidence and Mortality Worldwide for 36 Cancers in 185 Countries. CA Cancer J. Clin..

[2] Pietragalla A., Arcieri M., Marchetti C., Scambia G., Fagotti A. (2020). Ovarian Cancer Predisposition beyond BRCA1 and BRCA2 Genes. Int. J. Gynecol. Cancer.

[3] Hao J., Liu Y., Zhang T., He J., Zhao H., An R., Xue Y. (2021). Efficacy and Safety of PARP Inhibitors in the Treatment of Advanced Ovarian Cancer: An Updated Systematic Review and Meta-Analysis of Randomized Controlled Trials. Crit. Rev. Oncol. Hematol..

[4] Lord C.J., Ashworth A. (2017). PARP Inhibitors: Synthetic Lethality in the Clinic. Science.

[5] Zheng J., Devalaraja-Narashimha K., Singaravelu K., Padanilam B.J. (2005). Poly(ADP-Ribose) Polymerase-1 Gene Ablation Protects Mice from Ischemic Renal Injury. Am. J. Physiol.-Ren. Physiol..

[6] Molin G.Z.D., Westin S.N., Msaouel P., Gomes L.M., Dickens A., Coleman R.L. (2020). Discrepancy in Calculated and Measured Glomerular Filtration Rates in Patients Treated with PARP Inhibitors. Int. J. Gynecol. Cancer.

[7] Lazareth H., Delanoy N., Cohen R., Boissier E., Ayari H., Combe P., Crespel C., Mercadier-Riaz E., Karras A., Courbebaisse M. (2020). Nephrotoxicity Associated With Niraparib. Am. J. Kidney Dis..

[8] Gupta S., Hanna P.E., Ouyang T., Yamada K.S., Sawtell R., Wang Q., Katz-Agranov N., Feghali L., Krasner C.N., Bouberhan S. (2023). Kidney Function in Patients with Ovarian Cancer Treated with Poly (ADP-Ribose) Polymerase (PARP) Inhibitors. JNCI J. Natl. Cancer Inst..

[9] Ledermann J.A., Oza A.M., Lorusso D., Aghajanian C., Oaknin A., Dean A., Colombo N., Weberpals J.I., Clamp A.R., Scambia G. (2020). Rucaparib for Patients with Platinum-Sensitive, Recurrent Ovarian Carcinoma (ARIEL3): Post-Progression Outcomes and Updated Safety Results from a Randomised, Placebo-Controlled, Phase 3 Trial. Lancet Oncol..

[10] Friedlander M., Matulonis U., Gourley C., du Bois A., Vergote I., Rustin G., Scott C., Meier W., Shapira-Frommer R., Safra T. (2018). Long-Term Efficacy, Tolerability and Overall Survival in Patients with Platinum-Sensitive, Recurrent High-Grade Serous Ovarian Cancer Treated with Maintenance Olaparib Capsules Following Response to Chemotherapy. Br. J. Cancer.

[11] Mirza M.R., Benigno B., Dørum A., Mahner S., Bessette P., Barceló I.B., Berton-Rigaud D., Ledermann J.A., Rimel B.J., Herrstedt J. (2020). Long-Term Safety in Patients with Recurrent Ovarian Cancer Treated with Niraparib versus Placebo: Results from the Phase III ENGOT-OV16/NOVA Trial. Gynecol. Oncol..

[12] Wu X.H., Zhu J.Q., Yin R.T., Yang J.X., Liu J.H., Wang J., Wu L.Y., Liu Z.L., Gao Y.N., Wang D.B. (2021). Niraparib Maintenance Therapy in Patients with Platinum-Sensitive Recurrent Ovarian Cancer Using an Individualized Starting Dose (NORA): A Randomized, Double-Blind, Placebo-Controlled Phase III Trial. Ann. Oncol..

[13] González-Martín A., Pothuri B., Vergote I., DePont Christensen R., Graybill W., Mirza M.R., McCormick C., Lorusso D., Hoskins P., Freyer G. (2019). Niraparib in Patients with Newly Diagnosed Advanced Ovarian Cancer. N. Engl. J. Med..

[14] Pujade-Lauraine E., Ledermann J.A., Selle F., Gebski V., Penson R.T., Oza A.M., Korach J., Huzarski T., Poveda A., Pignata S. (2017). Olaparib Tablets as Maintenance Therapy in Patients with Platinum-Sensitive, Relapsed Ovarian Cancer and a BRCA1/2 Mutation (SOLO2/ENGOT-Ov21): A Double-Blind, Randomised, Placebo-Controlled, Phase 3 Trial. Lancet Oncol..

[15] Li N., Zhu J., Yin R., Wang J., Pan L., Kong B., Zheng H., Liu J., Wu X., Wang L. (2023). Treatment With Niraparib Maintenance Therapy in Patients With Newly Diagnosed Advanced Ovarian Cancer. JAMA Oncol..

[16] Monk B.J., Parkinson C., Lim M.C., O’Malley D.M., Oaknin A., Wilson M.K., Coleman R.L., Lorusso D., Bessette P., Ghamande S. (2022). A Randomized, Phase III Trial to Evaluate Rucaparib Monotherapy as Maintenance Treatment in Patients With Newly Diagnosed Ovarian Cancer (ATHENA-MONO/GOG-3020/ENGOT-Ov45). J. Clin. Oncol..

[17] Li N., Zhang Y., Wang J., Zhu J., Wang L., Wu X., Yao D., Wu Q., Liu J., Tang J. (2022). Fuzuloparib Maintenance Therapy in Patients With Platinum-Sensitive, Recurrent Ovarian Carcinoma (FZOCUS-2): A Multicenter, Randomized, Double-Blind, Placebo-Controlled, Phase III Trial. J. Clin. Oncol..

[18] Ren X., Sun P., Wang Y. (2024). PARP Inhibitor-Related Acute Renal Failure: A Real-World Study Based on the FDA Adverse Event Reporting System Database. Expert Opin. Drug Saf..

[19] Devi S., Chandrababu R. (2025). Impact of Olaparib, Niraparib, Rucaparib Therapies on Newly Diagnosed and Relapsed Ovarian Cancer -Systematic Review and Meta-Analysis. Asian Pac. J. Cancer Prev. APJCP.

[20] Ray-Coquard I., Pautier P., Pignata S., Pérol D., González-Martín A., Berger R., Fujiwara K., Vergote I., Colombo N., Mäenpää J. (2019). Olaparib plus Bevacizumab as First-Line Maintenance in Ovarian Cancer. N. Engl. J. Med..

[21] Petousis S., Kahramanoglu I., Appenzeller-Herzog C., Angeles M.A., Margioula-Siarkou C., Kacperczyk-Bartnik J., Bilir E., Chatzakis C., Caruso G., Bizzarri N. (2025). PARP Inhibitor Maintenance After First-Line Chemotherapy in Advanced-Stage Epithelial Ovarian Cancer: A Systematic Review and Meta-Analysis. JAMA Netw. Open.

[22] Li X., Li Z., Ma H., Li X., Zhai H., Li X., Cheng X., Zhao X., Zhao Z., Hao Z. (2024). Ovarian Cancer: Diagnosis and Treatment Strategies (Review). Oncol. Lett..

[23] Wang X., Mu J., Ma K., Ma Y. (2024). Challenges of Serum Creatinine Level in GFR Assessment and Drug Dosing Decisions in Kidney Injury. Adv. Pharm. Bull..

[24] Nakada T., Kudo T., Ito K. (2023). Quantitative Consideration of Clinical Increases in Serum Creatinine Caused by Renal Transporter Inhibition. Drug Metab. Dispos..

[25] Morrissey K.M., Stocker S.L., Wittwer M.B., Xu L., Giacomini K.M. (2013). Renal Transporters in Drug Development. Annu. Rev. Pharmacol. Toxicol..

[26] Zamek-Gliszczynski M.J., Sangha V., Shen H., Feng B., Wittwer M.B., Varma M.V.S., Liang X., Sugiyama Y., Zhang L., Bendayan R. (2022). Transporters in Drug Development: International Transporter Consortium Update on Emerging Transporters of Clinical Importance. Clin. Pharmacol. Ther..

[27] Jin Y.-W., Ma Y.-R., Liu Y.-T., Yang J.-R., Zhang M.-K., Ran F.-L., Chen Y., Wu X.-A. (2024). Identification of a Substrate of the Renal Tubular Transporters for Detecting Drug-Induced Early Acute Kidney Injury. Toxicol. Sci..

[28] Mathialagan S., Chung G., Pye K., Rodrigues A.D., Varma M.V.S., Brown C. (2024). Significance of Organic Anion Transporter 2 and Organic Cation Transporter 2 in Creatinine Clearance: Mechanistic Evaluation Using Freshly Prepared Human Primary Renal Proximal Tubule Cells. J. Pharmacol. Exp. Ther..

[29] Sponfeldner M.I., Andrikyan W., Maas R., Fromm M.F. (2024). Pseudo-Worsening of Kidney Function Due to Inhibition of Renal Creatinine Secretion: Quality of Information Provided in Prescribing Information/SmPC. Clin. Pharmacol. Ther..

[30] Mathialagan S., Rodrigues A.D., Feng B. (2017). Evaluation of Renal Transporter Inhibition Using Creatinine as a Substrate In Vitro to Assess the Clinical Risk of Elevated Serum Creatinine. J. Pharm. Sci..

[31] Bruin M.A.C., Sonke G.S., Beijnen J.H., Huitema A.D.R. (2022). Pharmacokinetics and Pharmacodynamics of PARP Inhibitors in Oncology. Clin. Pharmacokinet..

[32] Zhao D., Long X., Wang J. (2023). Transporter-mediated Drug-drug Interactions Involving Poly (ADP-ribose) Polymerase Inhibitors (Review). Oncol. Lett..

[33] Deng F., Sistonen J., Neuvonen M., Niemi M. (2023). Inhibition of Efflux Transporters by Poly ADP-Ribose Polymerase Inhibitors. Basic Clin. Pharmacol. Toxicol..

[34] Yamaoka K., Fujiwara M., Uchida M., Uesawa Y., Muroi N., Shimizu T. (2022). Comprehensive Analysis of Adverse Events Induced by PARP Inhibitors Using JADER and Time to Onset. Life.

